# Online Interventions Addressing Health Misinformation: Scoping Review

**DOI:** 10.2196/69618

**Published:** 2025-09-04

**Authors:** Hiya Grover, Radwa Nour, Nabil Zary, Leigh Powell

**Affiliations:** 1 School of Medicine Queen's University Belfast Belfast United Kingdom; 2 Institute of Learning Mohammed Bin Rashid University of Medicine and Health Sciences Dubai Health Dubai United Arab Emirates

**Keywords:** misinformation, disinformation, educational intervention, health, health care

## Abstract

**Background:**

Misinformation in health and health care contexts threatens public health by undermining initiatives, spreading dangerous behaviors, and influencing decision-making. Given its reach on online platforms and social media, there is growing demand for interventions addressing misinformation. Literature highlights the importance of theoretical underpinnings (frameworks and models) to guide the development of educational interventions targeting both the features of misinformation and the human traits that increase susceptibility.

**Objective:**

This review examines literature on online interventions targeting health misinformation to mitigate adverse public health impacts. It explores intervention types, population demographics, susceptibility-related human attributes, and misinformation characteristics addressed. It also identifies the theoretical underpinnings used and gaps in the literature.

**Methods:**

The review followed a methodological framework and adhered to PRISMA-ScR (Preferred Reporting Items for Systematic Reviews and Meta-Analyses extension for Scoping Reviews) guidelines. A search strategy combining Medical Subject Headings (MeSH) and keywords was used to search five databases for studies published between 2018 and 2024. Identified studies underwent deduplication, title and abstract screening using predefined eligibility criteria, full-text screening, and data extraction.

**Results:**

The initial search yielded 513 citations; 30 (5.8%) studies were included after screening. Of these, 19 (63%) focused on COVID-19 misinformation, 11 (37%) on other health contexts, and 1 (3%) addressed misinformation conceptually. Regarding intervention type, 22 (73%) used educational courses, 7 (23%) employed counter speech, and 1 (3%) used inoculation games, with some overlap. Sixteen (53%) interventions targeted characteristics of misinformation, categorized as content and presentation tactics, cognitive and psychological biases, social and cultural influences, and dissemination strategies. Seven (23%) interventions focused on specific demographics, while 14 (47%) addressed human attributes that heighten susceptibility. These attributes were grouped into knowledge and processing, emotional and psychological factors, and trust and social dynamics. Theoretical underpinnings guided intervention development in 23 (77%) studies, often overlapping in categories including inoculation and correction, education and cognition, motivation and emotion, behavior and persuasion, trust and belief, and learning design.

**Conclusions:**

Online interventions targeting health misinformation often share outcome goals and use overlapping strategies such as educational courses, counter speech, and inoculation games. Many adopt multifaceted approaches to address misinformation’s complexity. However, gaps remain in tailoring interventions to misinformation characteristics that could improve specificity and impact. Few studies focus on human attributes contributing to belief in and spread of misinformation, particularly among vulnerable groups. While theoretical models are commonly cited, clearer reporting and stronger connections to intervention design are needed. Collaboration among intervention developers, theorists, and psychologists is recommended to enhance future interventions.

**International Registered Report Identifier (IRRID):**

RR2-10.31219/osf.io/mfujb

## Introduction

### Background

Misinformation is defined as “false information that is spread, regardless of whether there is intent to mislead” [1]. Misinformation in health and health care contexts has emerged as a significant and growing concern, particularly in the digital age where the spread of information is rapid and often unchecked. According to the World Health Organization (WHO), health misinformation is a global threat, and due to its rapid online dissemination, it undermines public health initiatives and propagates dangerous health-related behaviors [2]. This issue has become increasingly pronounced during public health crises, such as the COVID-19 pandemic, when individuals are more likely to seek information online [3]. The WHO further asserts that, in times of a public health crisis, the risks and consequences resulting from misinformation can be equivalent to those of the health crisis itself, primarily due to confusion and misinformed decision-making [4].

Both historically and in the present, the significance of health misinformation has been a growing cause for concern about public health outcomes; for example, misinformation about vaccination conspiracies and alleged links to autism has negatively affected vaccine uptake and contributed to localized measles outbreaks [5]. During the Ebola outbreaks, misinformation suggesting that the disease was not real led to reduced acceptance of formal health care services, including vaccination and hospital attendance, thereby facilitating further spread of the virus [6]. More recently, COVID-19 misinformation placed an increased burden on public health, leading individuals to disregard social distancing rules and mask-wearing precautions and instead rely on non–evidence-based treatments [7].

Many factors can cause a person to fall prey to misinformation. Misinformation often possesses intrinsic characteristics, specific qualities, or features embedded into the content that increase the audience’s belief in it and their tendency to disseminate it further. These characteristics include, but are not limited to, emotional appeal, repetition, use of fake experts, and vagueness [8]. Research has shown that individuals are also prone to misinformation due to human attributes, namely natural cognitive tendencies, knowledge gaps, and psychological shortcuts, which misinformation exploits, thereby impacting their belief systems and decision-making processes [8].

Misinformation is disseminated through a multitude of channels, including social media, online communication groups, traditional media, and word of mouth. With the increasing use of the internet and social media, the speed of dissemination has risen due to the underlying architecture that enables coordinated efforts or viral patterns to drive spread [9]. Its reach is faster and broader on social media, given its sensationalist and emotional nature, which increases individuals’ susceptibility and likelihood of uptake.

Health misinformation shares some attributes with other categories of misinformation. Certain attributes are ubiquitous and must be targeted in all intervention designs. These attributes include those stemming from scientific skepticism, influenced by human attributes such as political ideology and sociocultural world views; repetition and amplification online; and the spread of false conspiracies and their impacts on decision-making [10]. However, other characteristics and human susceptibility attributes are unique to health misinformation and must be considered in intervention development. These include the lack of trust in health professionals, government agencies, and pharmaceutical companies; the polarizing use of emotions; and religious framing [11].

Interventions to address health misinformation include peer-to-peer debunking, seminars, educational courses about news literacy, and tools to identify fake news [12]. A subcategory of these are online interventions, which have a broad and diverse reach compared to conventional or face-to-face approaches, allowing for the rapid dissemination of information to all populations, including those that typically do not engage with traditional health care systems. With the growing use of the internet for health-related information, online interventions are particularly beneficial in addressing the root causes of misinformation [13]. Given the widespread nature of misinformation in digital spaces, online interventions are adaptable resources that have been used to deliver real-time corrections based on news alerts, provide health updates based on public health agency announcements, implement algorithmic corrections, and promote digital health literacy through educational courses in an environment familiar to users [13].

Despite the growing body of research exploring factors that contribute to the spread and acceptance of misinformation in health and health care contexts, there remains a knowledge gap in how to design interventions to combat these factors. Research has demonstrated that individuals are prone to misinformation due to intrinsic factors (eg, cognitive biases) or extrinsic factors (eg, social influences and cultural norms), ultimately impacting belief systems and decision-making processes. Misinformation also has characteristics that enhance its spread and impact, such as emotional appeal, oversimplification, and false authority. Despite increasing efforts to design interventions targeting health misinformation, many do not adequately discuss or address the characteristics of misinformation that put audiences at risk of believing and disseminating it; for example, an online before-and-after intervention study by Duarte et al [14] focused on fact-checking claims circulating in Brazil about the health benefits of coconut oil consumption. While the intervention addressed factual accuracy, it did not address intrinsic features of the misinformation, such as the use of false experts, celebrity endorsements, repetition, or cultural beliefs.

Similarly, other studies do not discuss how their interventions address the human attributes that make it easy for individuals to be targeted by misinformation; for example, an intervention by Pennycook et al [15] assessed how misinformation is more believable and easier to disseminate when presented on social media platforms but did not address the human factors that make individuals susceptible to it, such as cultural norms, literacy levels, or system 1 thinking. Moreover, these interventions often lack theoretical guidance in their design and implementation; as noted by Kreuter et al [16], theoretical frameworks and models should serve as fundamental guiding elements to develop and implement successful interventions. Without a deeper understanding of these shortcomings, there is a risk that future interventions will continue to fall short in their effectiveness against the rapidly evolving threats posed by health misinformation.

This scoping review aims to synthesize an understanding of the characteristics used in the development of online interventions to address misinformation in health and health care contexts. We have mapped existing literature to understand the theoretical underpinnings that guide the development of interventions targeting both the characteristics of misinformation and the human attributes that make audiences susceptible to it. This scoping review will be useful for health care professionals, policy makers, and public health organizations, as it will provide insights into effective strategies for countering misinformation. By understanding the theoretical underpinnings and characteristics that drive both misinformation and susceptibility to it, stakeholders can design effective interventions that reach diverse populations, particularly those who rely on the internet for health-related information.

### Rationale

Current literature and reviews predominantly focus on interventions addressing misinformation related to specific health issues, such as COVID-19, autism, vaccine hesitance, and human papillomavirus [17,18]. However, there is a lack of reviews assessing interventions targeting misinformation broadly across diverse health contexts. Given the widespread impact of misinformation, a holistic approach is needed to guide the development of interventions addressing this root dilemma in health communication. While other scoping reviews have focused on interventions targeting misinformation on social media platforms [3,12], these represent only a subset of online misinformation. This scoping review aims to explore the full range of interventions addressing misinformation across all online platforms and contexts, providing a more comprehensive understanding of their impact. Although extensive theoretical and psychological analyses have explored how misinformation is formed, perceived, and perpetuated, particularly in relation to cognitive biases and social media dynamics, there is a notable absence of holistic reviews examining the scope of implemented interventions [19]. Consequently, there is insufficient insight into how theoretical frameworks, characteristics of misinformation, and demographic factors influence the development of these interventions. This scoping review aims to address these gaps by mapping the range of interventions targeting health misinformation and charting the integration of theoretical underpinnings and human attributes into their design.

Our rationale behind choosing a scoping review methodology is to examine the breadth of literature on this topic, understand the themes and theoretical underpinnings (including frameworks and models) used to develop online interventions, and enable conceptual mapping [20]. As our objective is to identify concepts and frameworks guiding intervention development rather than to assess study quality or outcome efficacy, a scoping review is the appropriate methodology.

### Objectives

This scoping review aims to identify existing literature on online interventions implemented to address health misinformation. The review will collate the theoretical underpinnings used in the development and design of interventions and examine how they address both the characteristics of misinformation and the human attributes of target populations, with the aim of preventing or mitigating the detrimental consequences of misinformation. This review addresses the following research questions:

1. What online interventions have been implemented to address misinformation in health and health care contexts to mitigate adverse effects on public health outcomes? (1.1) What types of interventions have been implemented? (1.2) What are the demographics of the target populations? (1.3) What human attributes are targeted in the intervention design? (1.4) What characteristics, if any, of misinformation do the interventions address?
2. What are the theoretical underpinnings used to develop and design these interventions?
3. What gaps in the literature are identified through this review?

## Methods

### Scoping Review Methodology

This scoping review followed the five-stage methodological framework provided by Arksey and O’Malley [21]: (1) identifying the research question; (2) identifying relevant studies; (3) study selection; (4) charting the data; and (5) collating, summarizing, and reporting the results. We report our results using the PRISMA-ScR (Preferred Reporting Items for Systematic Reviews and Meta-Analyses extension for Scoping Reviews) guidelines. An in-depth protocol detailing the search strategies, study selection, and screening was published in 2024 [22].

### Information Sources, Search, Selection of Sources of Evidence, and Eligibility Criteria

The search strategy began with a preliminary scan of Google Scholar to identify relevant concepts in both gray and peer-reviewed literature, after which a detailed list of keywords and Medical Subject Headings (MeSH) terms was developed with the support of a library technical expert (Multimedia Appendix 1). A search strategy was then created to target 3 key concepts: “health,” “misinformation,” and “online intervention.” This strategy was first tested on PubMed and refined using benchmark studies. Search strings were generated using the keywords, Boolean operators, and limiters to ensure sensitivity, precision, and relevance. These search strings were then implemented for an electronic bibliographic search in 5 databases: PubMed, MEDLINE, PsycInfo, Scopus, and the Cochrane Library. An initial search was completed at the time of protocol development [22] in June 2023; however, to ensure comprehensiveness and relevance, the search was repeated in June 2024.

The search included English-language studies published between 2018 and 2024. This time frame was used to maintain temporal relevance to the recent trends and topography of misinformation and interventions in health and health care contexts. By 2018, online platforms, including social media, had gained significant traction as sources of health information, marking a turning point for studying misinformation trends [3]. Prepandemic trends were relevant to the review, as misinformation (eg, antivaccine sentiments and medical skepticism) was already increasing before the pandemic [23]. In addition, artificial intelligence–driven tools and online algorithms were rapidly evolving during this period, exponentially contributing to the misinformation infodemic, which required targeted interventions [24]. To capture a baseline of how misinformation interventions evolved before, during, and after the acute phase of the pandemic across different health contexts, we selected a publication window from 2018 to 2024.

All relevant citations from the databases were uploaded to Zotero (version 6.0.2; Corporation for Digital Scholarship), where duplicates were removed. The deduplicated citations were then exported to the Rayyan platform (Rayyan Systems Inc) for title and abstract screening by two independent reviewers. Articles were excluded if they were undetected duplicates or did not meet the eligibility criteria (Textbox 1). Any discrepancies were resolved through discussion and with input from a third reviewer to achieve consensus. To be included in full-text screening and data extraction, the citations had to meet the predefined eligibility criteria.

**Inclusion and exclusion criteria.**

**Inclusion criteria**

- Interventions presented, delivered, and disseminated on online platforms
- Studies directly addressing misinformation in health and health care contexts
- Interventional studies with an experimental study design and methodology
- Papers published in English

**Exclusion criteria**

- Studies using offline, face-to-face, or in-person forms of intervention presentation or delivery
- Studies addressing misinformation unrelated to health (eg, climate change or politics)
- Noninterventional, nonexperimental studies (eg, observational or theoretical studies)
- Papers published in languages other than English

Peer-reviewed journal articles, published conference papers, and work-in-progress papers were eligible for data extraction. Systematic and scoping reviews identified in the search were excluded.

### Data-Charting Process

After title and abstract screening, two reviewers independently screened the full-text papers for inclusion based on the predefined criteria. Any discrepancies were addressed through discussion and consensus, with input from a third reviewer. This process yielded 30 papers. Data were extracted from each paper by one reviewer, with a second reviewer independently extracting data from a subset of the papers (6/30, 20%) for alignment. Data extraction was performed using Microsoft Forms (Multimedia Appendix 2) and included the following: intervention type, intervention content, mode of delivery, demographics of the target populations, human attributes targeted, theoretical underpinnings used to develop the intervention, and characteristics of the misinformation targeted.

The types of intervention were categorized into 5 groups based on preliminary research and screening (Textbox 2 [25-29]). This categorization was represented as a drop-down menu in the extraction form.

**Intervention-type categories.**

1. Educational courses: a structured and systematic approach designed to impart knowledge, skills, attitudes, or behaviors to a specific target audience, typically in the format of written or video learning modules or courses, fact-checking training, peer-driven learning and teaching, or integrated discussion platforms; aimed at improving understanding, changing perceptions, or promoting specific actions [25,26]
2. Inoculation games: game-based delivery of inoculation, that is, exposure to “weakened” doses of misinformation, along with guidance on identification, correction, and information, to “prebunk” or preemptively educate individuals against future encounters with misinformation; with elements of gamification, including, but not limited to, debate, simulation, identification, narrative, and cognitive bias training games [27]
3. Counter speech: a strategy used to combat misinformation by directly addressing and refuting it, involving providing accurate information, promoting critical thinking, and offering alternative perspectives to undermine the credibility and influence of misinformation; examples include, but are not limited to, myth debunking, fact checking, expert testimonials, personal stories, humor, satire, call to action, questioning source credibility, encouraging critical thinking, and highlighting consequences [28]
4. Online intervention distribution blockers: technology-based tools designed to prevent or limit the spread of specific types of content (namely misinformation) online; examples include, but are not limited, to advertisement blockers, content filters, social media blockers, email spam blockers, content-blocking domain name system servers, and comment moderation tools [29]
5. Other: interventions that do not fit into the aforementioned categories, including, but not limited to, those based on artificial intelligence, virtual reality, augmented reality, and online community-based discussion networks

### Synthesis of Results

Charted data were exported to Microsoft Excel for preliminary refining and cleaning in preparation for further analysis. For numerical and binary data, results were tabulated using Excel formulas to calculate frequency counts and percentages.

The data were also qualitatively analyzed to identify themes that would support the differentiation and categorization of long, descriptive-type responses. MAXQDA (version 24.4.0; VERBI Software GmbH) was used for thematic qualitative analysis, paralleling the methodology formulated by Braun and Clarke [30]. Long responses from the data-charting table were exported and systematically analyzed by assigning word codes to recurring ideas, concepts, and themes. The reviewers grouped common themes based on the research questions, grouped them into categories and named them with assistance from generative artificial intelligence (ChatGPT with GPT-4, OpenAI [31]), and exported them to Excel for frequency analysis. As recommended by Braun and Clarke [30], a flexible approach incorporating both deductive and inductive coding was used for thematic analysis.

## Results

### Selection of Sources of Evidence

**Figure 1:**
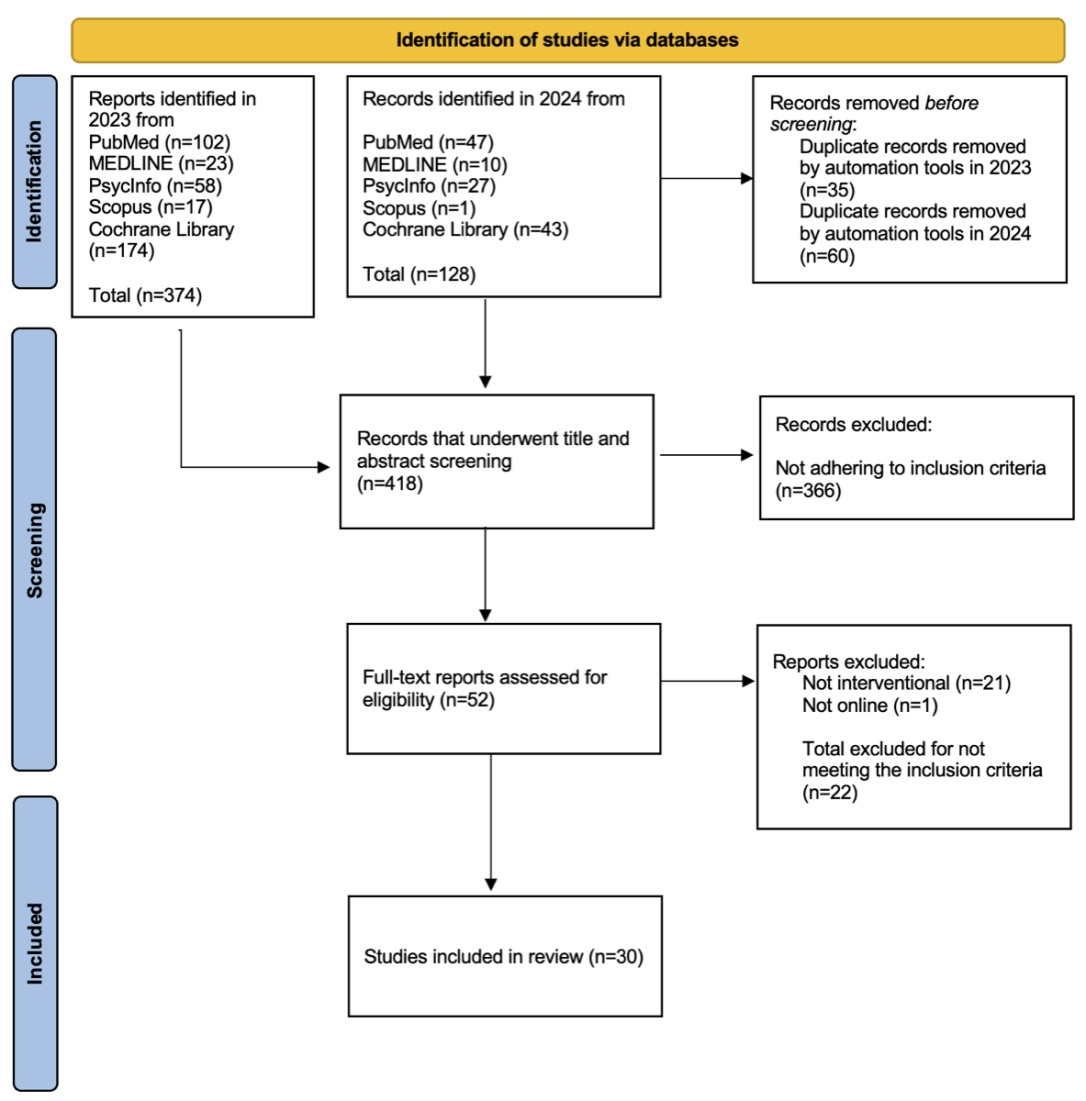
PRISMA-ScR (Preferred Reporting Items for Systematic Reviews and Meta-Analyses extension for Scoping Reviews) diagram.

Searches across the 5 databases yielded 513 citations; after the removal of duplicates, 418 (81.5%) articles underwent title and abstract screening. Of these 418 articles, 52 (12.4%) were deemed eligible for combined full-text screening and data extraction. After full-text screening, 30 (58%) of the 52 articles were included, and 22 (42%) were excluded. The PRISMA-ScR is diagram is shown in Figure 1. Reasons for exclusion were as follows: (1) not interventional (21/22, 95%) and (2) not online (1/22, 5%). A summary of study features, intervention types, and targeted misinformation characteristics is presented in Multimedia Appendix 3 [14,15,32-59].

Table 1 presents a list of the included studies along with their titles.

**Table 1:** Studies and study titles.

Study	Study title
Geana et al [32]	“A friendly conversation.” Developing an eHealth intervention to increase COVID-19 testing and vaccination literacy among women with criminal and legal system involvement
Powell et al [33]	A web-based public health intervention for addressing vaccine misinformation: protocol for analyzing learner engagement impacts on the hesitancy to vaccinate
Scales et al [34]	Addressing antivaccine sentiment on public social media forums through web-based conversations based on motivational interviewing techniques: observational study
Steffens et al [35]	Addressing myths and vaccine hesitancy: a randomised trial
Del Pozo et al [36]	Can touch this: training to correct police officer beliefs about overdose from incidental contact with fentanyl
Domgaard and Park [37]	Combating misinformation: the effects of infographics in verifying false vaccine news
Ecker et al [38]	Correcting vaccine misinformation: a failure to replicate familiarity or fear-driven backfire effects
Winters et al [39]	Debunking highly prevalent health misinformation using audio dramas delivered by WhatsApp: evidence from a randomised controlled trial in Sierra Leone
Lu et al [40]	Does public fear that bats spread COVID-19 jeopardize bat conservation?
Alsaad and AlDossary [41]	Educational video intervention to improve health misinformation identification on WhatsApp among Saudi Arabian population: pre-post intervention study
Ugarte and Young [42]	Effects of an online community peer-support intervention on COVID-19 vaccine misinformation among essential workers: mixed-methods analysis
Iles et al [43]	Effects of narrative messages on key COVID-19 protective responses: findings from a randomized online experiment
Abroms et al [44]	Empathic engagement with the COVID-19 vaccine hesitant in private Facebook groups: a randomized trial
Koban [45]	Empathic engagement with the vaccine hesitant in online spaces
Beleites et al [46]	Evaluating the impact of short animated videos on COVID-19 vaccine hesitancy: an online randomized controlled trial
Paynter et al [47]	Evaluation of a template for countering misinformation—real-world autism treatment myth debunking
Pennycook et al [15]	Fighting COVID-19 misinformation on social media: experimental evidence for a scalable accuracy-nudge intervention
Ma et al [48]	Fighting COVID-19 misinformation through an online game based on the inoculation theory: analyzing the mediating effects of perceived threat and persuasion knowledge
Gavin et al [49]	Fighting the spread of COVID-19 misinformation in Kyrgyzstan, India, and the United States: how replicable are accuracy nudge interventions?
Sundstrom et al [50]	HPV vaccination champions: evaluating a technology-mediated intervention for parents
Jiang et al [51]	Inoculation works and health advocacy backfires: building resistance to COVID-19 vaccine misinformation in a low political trust context
Agley et al [52]	Intervening on trust in science to reduce belief in COVID-19 misinformation and increase COVID-19 preventive behavioral intentions: randomized controlled trial
van Stekelenburg et al [53]	Investigating and improving the accuracy of US citizens’ beliefs about the COVID-19 pandemic: longitudinal survey study
Duarte et al [14]	Misinformation in nutrition through the case of coconut oil: an online before-and-after study
MacFarlane et al [54]	Refuting spurious COVID-19 treatment claims reduces demand and misinformation sharing
van der Meer and Jin [55]	Seeking formula for misinformation treatment in public health crises: the effects of corrective information type and source
Piltch-Loeb et al [56]	Testing the efficacy of attitudinal inoculation videos to enhance COVID-19 vaccine acceptance: quasi-experimental intervention trial
Vandormael et al [57]	The effect of a wordless, animated, social media video intervention on COVID-19 prevention: online randomized controlled trial
Vraga et al [58]	The effects of a news literacy video and real-time corrections to video misinformation related to sunscreen and skin cancer
Tanemura and Chiba [59]	The usefulness of a checklist approach-based confirmation scheme in identifying unreliable COVID-19-related health information: a case study in Japan

### Characteristics of Sources of Evidence

Of the 30 studies published between 2018 and 2024, the period between 2018 and 2020 saw 3 (10%) studies published; 11 (37%) studies were published in 2021; in 2022, a total of 6 (20%) studies were published; 2023 saw the publication of 8 (27%) studies; and 2 (7%) studies were published in 2024.

Geographic variation was evident. Of the 30 studies, 2 (7%) were conducted in >1 country; moreover, 20 (67%) were conducted in the United States [32,34,36,37,42-50,52-58], 2 (7%) in Australia [35,47], 2 (7%) in the United Kingdom [38,57], and 1 (3%) each in China [40], Brazil [14], Sierra Leone [39], Hong Kong [51], India [49], Kyrgyzstan [49], Japan [59], Saudi Arabia [41], Germany [57], Spain [57], and Mexico [57]; and United Arab Emirates [33].

### Study Designs

All included studies were interventional in nature. The most common design was randomized controlled trials (20/30, 67%) [15,35,37-40,42,43,45-49,51-55,57,58]. Of the 30 studies, 1 (3%) described its design as a quasi-experimental intervention trial [56]. In addition, 2 (7%) of the 30 studies used single-arm posttest-only designs [34,44], and 7 (23%) used single-arm pretest-posttest designs [14,32,33,36,41,50,59].

### Types of Interventions

Of the 30 included studies, 22 (73%) used educational courses [15,32,33,37-43,46,47,49-53,55-59]; among these, 2 (9%) incorporated counter speech in ways that could not be considered mutually exclusive from the educational component [42,50]. Counter speech alone was used in 7 (23%) of the 30 studies [14,34-36,44,45,54], while 1 (3%) study used an inoculation game as the intervention [48].

### Content and Delivery

#### Misinformation Targeted

All studies targeted misinformation in health and health care contexts. Of the 30 studies, 19 (63%) addressed COVID-19–related misinformation [15,32-34,40-46,48,49,51-53,56,57,59]; 11 (37%) addressed other health misinformation, including claims about sunscreen and skin cancer, non–COVID-19 vaccines, drug overdose, malaria, typhoid, human papillomavirus, and autism [14,35-39,47,50,54,55,58]; and 1 (3%) targeted misinformation in general, focusing on techniques for identifying misinformation on social media [41].

#### Digital Content

The intervention content primarily included text and multimedia elements such as audio, videos, images, and graphics. Of the 30 interventions, 9 (30%) used multimedia only [32,36,37,39,41,50,52,56,57]; 9 (30%) used text only [14,34,35,43,45,51,54,55,59]; and 12 (40%) used >1 type of digital content, such as combinations of text, multimedia, infographics, focus groups, and online discussion platforms [15,33,38,40,42,44,46-49,53,58].

#### Mode of Delivery

Websites were used for delivery in 22 (73%) of the 30 studies [14,15,32,33,35,37,38,41,43,46-49,51-59]. Of these 22 studies, 1 (5%) used an online course development platform [33], 1 (5%) used a web-based eHealth application [32], and 15 (68%) used web-based or online questionnaire or survey platforms (eg, Qualtrics and Cross Marketing Inc) [14,15,35,37,38,41,43,48,49,51-54,56,59]. Of the 22 studies, 1 (5%) used Zoom [36], 1 (5%) used emailing platforms alongside social media [50], and 7 (32%) used social media [34,39,40,42,44,45,50]. Of these 7 interventions, 5 (71%) used Facebook [34,42,44,45,50], 1 (14%) used WhatsApp [39], and 1 (14%) used WeChat [40].

### Targeted Characteristics of Misinformation

A range of characteristics of misinformation was addressed across the studies. Of the 30 studies, 15 (50%) [15,35-37,40-43,47-49,52,54,56,59] reported that their interventions targeted specific characteristics of misinformation, while the remaining studies (n=14, 47%) either did not include these elements or did not report them. Of the 16 studies, 9 (56%) reported that the intervention targeted >1 characteristic [35,37,40,42,45,47,48,54,56].

**Figure 2:**
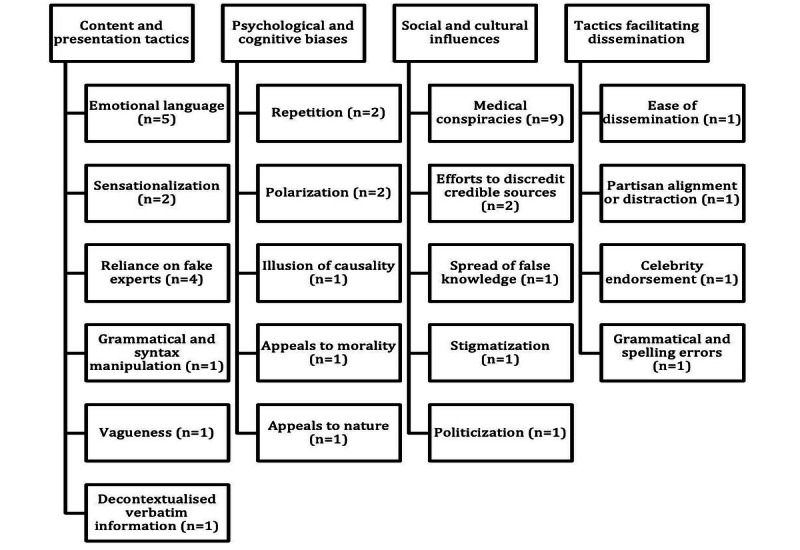
Hierarchy diagram grouping the targeted characteristics of misinformation into four categories: (1) content and presentation tactics, (2) psychological and cognitive biases, (3) social and cultural influences, and (4) tactics facilitating dissemination (“n” indicates the number of studies reporting each characteristic).

These targeted characteristics were grouped into four broad categories: (1) content and presentation tactics, (2) psychological and cognitive biases, (3) social and cultural influences, and (4) tactics facilitating dissemination (Figure 2).

Content and presentation tactics refer to elements that influence the audience’s perception, interpretation, and emotional response to misinformation, with the goal of enhancing the persuasiveness or impact of the content. These included the use of emotional language (5/16, 31%) [37,40,47,48,56]; sensationalization (2/16, 13%) [37,40]; reliance on fake experts (4/16, 25%) [37,47,48,54]; and the presence of grammatical and syntax manipulation (1/16, 6%) [40], vagueness (1/16, 6%) [37], and decontextualized verbatim factual information (1/16, 6%) [45].

Psychological and cognitive biases refer to systematic patterns of deviation from norms or rationality in judgment that affect perception, memory, thought, and decision-making abilities. These biases often unconsciously influence beliefs, attitudes, and behaviors through heuristic shortcuts biases and are exacerbated by emotional and social influences. Reported examples included repetition (2/16, 13%) [45,47], polarization (2/16, 13%) [48,54], and the illusion of causality (1/16, 6%) [54], alongside appeals to morality (1/16, 6%) [54] and nature (1/16, 6%) [54].

Social and cultural influences refer to how the manipulation of societal norms, cultural beliefs, and community behaviors affects individuals’ perceptions, attitudes, and actions, shaping how they interpret misinformation, form opinions, and make decisions in health and health care contexts. These influences encompassed medical conspiracies (9/16, 56%) [35,36,40,43,47,48,52,54,56]; efforts to discredit credible sources (2/16, 13%) [48,54]; and the spread of false knowledge (1/16, 6%) [48], often through stigmatization (1/16, 6%) [42] and politicization (1/16, 6%) [42].

Tactics facilitating dissemination are strategies that enhance the spread and reach of misinformation—making it easier for content to be shared widely and rapidly across various platforms—by exploiting the characteristics of digital media, social networks, and human psychology. These tactics included ease of dissemination (1/16, 6%) [49], partisan alignment or distraction (1/16, 6%) [15], celebrity endorsement (1/16, 6%) [47], and grammatical and spelling errors (1/16, 6%) [37].

The study by Tanemura and Chiba [59] specifically postulated the use of a 5-step framework of confirmation theory, incorporating characteristics such as “source credibility, scientific evidence, consistency, bias identification, transparency,” which parallel elements found in other interventions.

The study by Ma et al [48] addressed the 6 characteristics of misinformation proposed by Roozenbeek and van der Linden [60] (ie, conspiracy theories, polarization, impersonation, emotion, discrediting opponents, and trolling), adding an additional characteristic: “the spreading of false knowledge.”

### Population Characteristics Reported

The following subsections describe the demographic characteristics of the populations in the included studies as well as the specific attributes or behaviors being addressed by interventions targeting misinformation.

#### Demographics

Most of the studies (22/30, 73%) focused on the general public or did not report specific characteristics of their target populations [14,15,33,34,37-41,43,45,46,48,49,51-54,56-59]. A subset of this group (2/22, 9%) included university students who were not directly targeted by the interventions but were part of a convenience sample [14,51]. Specific demographics targeted in other studies included educators or teachers (2/30, 7%) [42,47]; health care workers (2/30, 7%) [42,47]; police officers (1/30, 3%) [36]; carers (1/30, 3%) [47]; and parents (2/30, 7%)—1 (50%) of these 2 studies focused on parents of children aged 0 to 5 years [35,50]. In addition, the study by Geana et al [32] targeted women involved with the criminal and legal justice system, while the study by Abroms et al [44] focused on individuals who were not vaccinated [44].

#### Human Attributes

**Figure 3:**
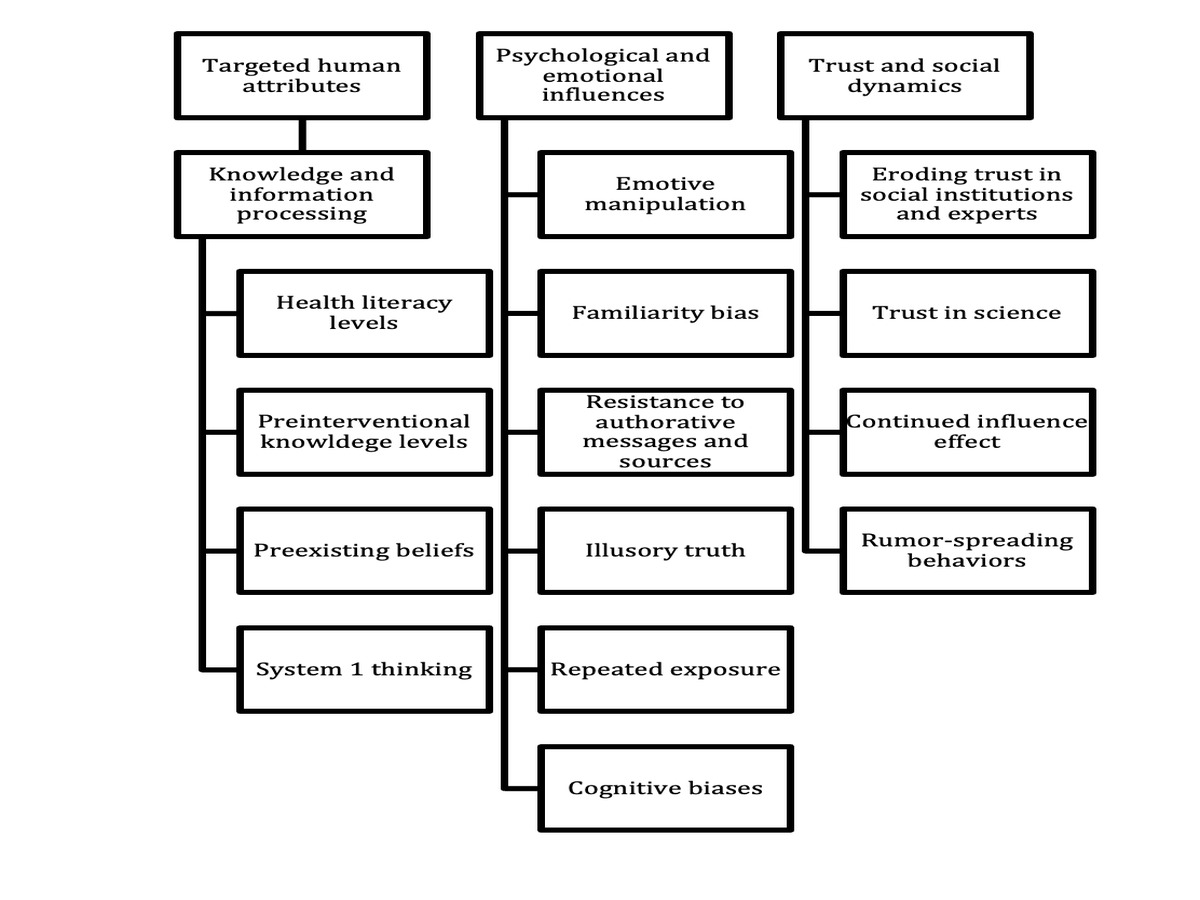
Hierarchy chart showing the human attributes targeted by the interventions of the included studies, categorized into (1) knowledge and information processing, (2) psychological and emotional influences, and (3) trust and social dynamics.

Of the 30 studies, 14 (47%) reported that their interventions targeted human attributes through design features [32,34,35,38-41,43,47,49,52-54,59]. These attributes—diverse factors influencing susceptibility to and perpetuation of misinformation across different contexts and populations—can be grouped into three categories: (1) knowledge and information processing, (2) psychological and emotional influences, and (3) trust and social dynamics. Of the 14 studies, 8 (57%) targeted >1 human attribute through their intervention design [32,38,39,41,43,47,49,54] (Figure 3).

Studies that targeted factors related to knowledge and information processing addressed health literacy levels (4/14, 29%) [32,41,53,59], preintervention knowledge levels (1/14, 7%) [40], the influence of preexisting beliefs (1/14, 7%) [54], and system 1 thinking (2/14, 14%) [49].

Psychological and emotional influences encompassed emotive manipulation (3/14, 21%) [41,43,47], familiarity bias (3/14, 21%) [35,38,43], resistance to authoritative messages and sources (2/14, 7%) [32,43], the concept of illusory truth (3/14, 21%) [34,38,43], repeated exposure (2/14, 7%) [39,47], and various cognitive biases (3/14, 21%) [39,49,54].

Trust and social dynamics were related to the continued influence effect (3/14, 21%) [38,39,47], eroding trust in social institutions and experts (1/14, 7%) [43], trust in science (1/14, 7%) [52], and behaviors such as rumor spreading (1/14, 7%) [41].

Geana et al [32] explicitly stated that the purpose and design of their intervention was to target the social determinants of health among women with criminal and legal system involvement, which made them more susceptible to misinformation.

The study by Tanemura and Chiba [59] targeted health literacy levels specifically among individuals in Japan with no medical background, a susceptibility factor associated with increased risk of misinformation uptake and dissemination.

### Theoretical Underpinnings of Interventions

#### Overview

**Figure 4:**
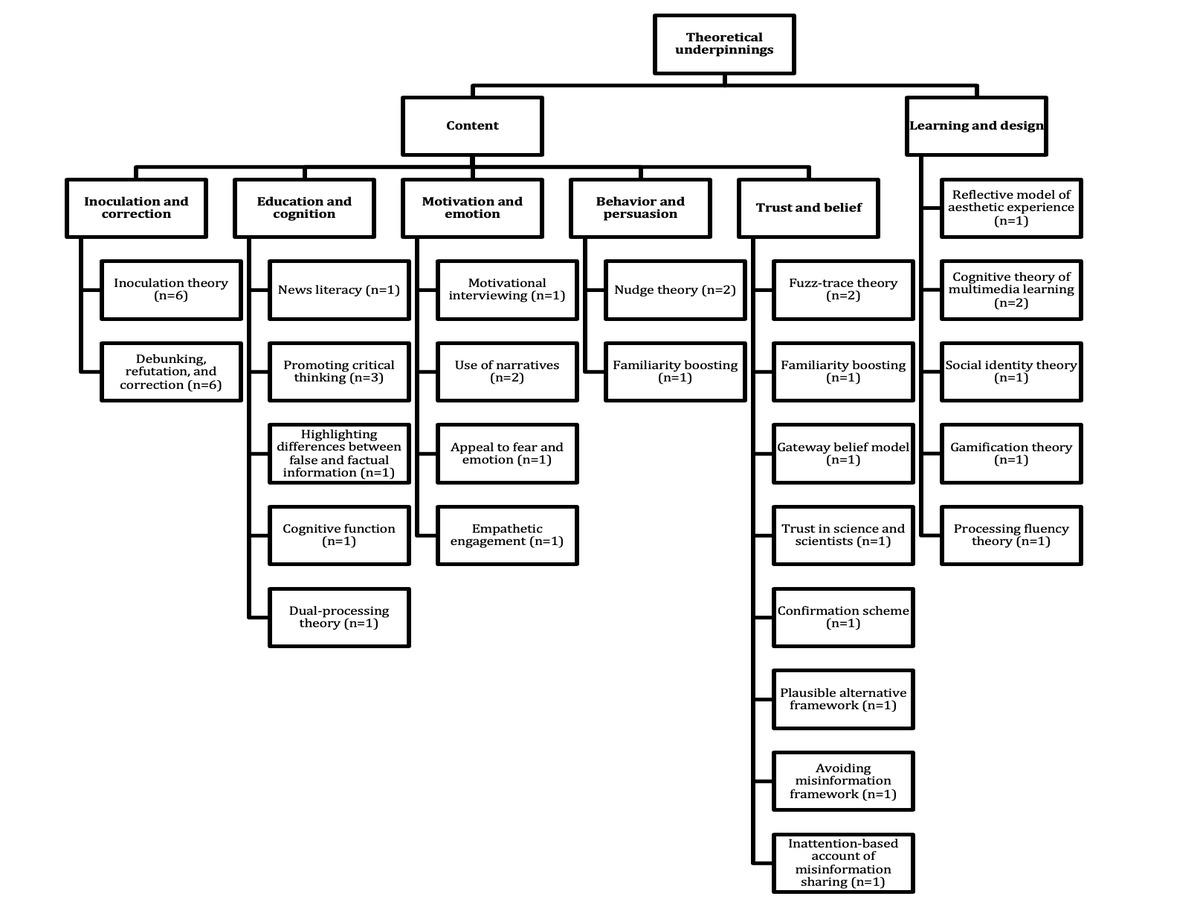
Hierarchy chart showing the theoretical underpinnings of the interventions described in the reviewed studies, categorized by elements of learning design and content. These are further classified into inoculation and correction, education and cognition, motivation and emotion, behavior and persuasion, and trust and belief (“n” indicates the number of studies reporting each type of theoretical underpinning).

Of the 30 studies, 23 (77%) reported the use of theoretical underpinnings to develop the content or learning and design component of the intervention [15,32-35,37-39,41,43-45,47-49,51-56,58,59]. Of these 23 studies, 12 (52%) used >1 theoretical underpinning [15,32,34,38,39,41,44,48,49,53,58,59]. The findings from these 23 studies, with considerable overlap, are grouped into the categories outlined in the following subsections (Figure 4).

#### Content

The category “inoculation and correction” included inoculation theory (6/23, 26%) [34,41,48,51,56,58] and debunking, refutation, and correction (6/23, 26%) [35,39,44,47,54,55,58]. The number of studies using some form of inoculation alongside correction techniques was substantial.

The category “education and cognition” encompassed news literacy (1/23, 4%) [58], promoting critical thinking (3/23, 13%) [34,49,53], highlighting differences between false and factual information (1/23, 4%) [54], cognitive function (1/23, 4%) [49], and dual-processing theory (1/23, 4%) [49].

The category “motivation and emotion” covered motivational interviewing (1/23, 4%) [34], the use of narratives (2/23, 9%) [34,43], appealing to fear and emotions (1/23, 4%) [38], and empathetic engagement (1/23, 4%) [44].

The category “behavior and persuasion” included nudge theory (2/23, 9%) [15,49], and boosting approach (1/23, 4%) [53].

The category “trust and belief” included fuzzy-trace theory (2/23, 9%) [44,45], familiarity boosting (1/23, 4%) [38], gateway belief model (1/23, 4%) [53], trust in science and scientists (1/23, 4%) [52], confirmation scheme (1/23, 4%) [59], plausible alternative framework (1/23, 4%) [39], avoiding misinformation framework (1/23, 4%) [39], and inattention-based account of misinformation sharing (1/23, 4%) [15].

#### Learning Design

Of the 24 studies that referenced theoretical underpinnings, 4 (17%) also reported the use of those that contributed to the learning and design aspect of the intervention [32,33,37,41]. These included reflective models of aesthetic experience (1/4, 25%) [32], the cognitive theory of multimedia learning (2/4, 50%) [32,41], social identity theory (1/4, 25%) [32], gamification theory (1/4, 25%) [33], and processing fluency theory (1/4, 25%) [37].

## Discussion

### Principal Findings

This review identified 30 studies describing online interventions implemented to address health misinformation between 2018 and 2024. The publication time frames of the included studies varied considerably but closely aligned with the COVID-19 pandemic and the associated infodemic. The period from 2021 to 2023 was marked by a significant surge in misinformation awareness, the dissemination of misinformation, and the urgency of the public health crisis. Despite the prominence of COVID-19–related studies, this review highlights a broader body of literature addressing misinformation in various health-related contexts. Notably, 40% (12/30) of the included papers focused on misinformation either before or after the COVID-19 pandemic. Although there was geographic variation across the included studies, the majority were conducted in the United States (20/30, 67%), indicating limited geographic representation overall.

Some of the interventions (14/30, 47%) considered a broad spectrum of human attributes, which we categorized into three groups based on cognitive and behavioral similarities: (1) knowledge and information processing, (2) psychological and emotional influences, and (3) trust and social dynamics. However, 16 (53%) of the 30 studies did not report or consider specific human attributes in their intervention designs. Furthermore, many of the interventions (23/30, 77%) lacked specificity in targeting population demographics, often defaulting to “general population” approaches. These gaps highlight limitations in designing population-specific interventions.

A varied selection of intervention types was identified in the review. Educational interventions addressing health misinformation included diverse formats, such as courses, videos, textual content, game-based learning modules, and infographics. A frequently used intervention strategy was counter speech (9/30, 30%), which involves directly addressing misinformation by highlighting inaccuracies and providing correct information. Inoculation games were used independently in some of the studies (1/30, 3%); however, the technique of inoculation (ie, preemptively exposing individuals to weakened forms of misinformation to build resistance) was often embedded within both educational interventions and counter-speech strategies. Several other studies highlighted the significance and potential of online intervention distribution blockers in mitigating health misinformation; however, these were not implemented in interventional or experimental study designs [12]. The interventions reviewed in this study addressed various characteristics of misinformation, with slightly more than half of the studies (16/30, 53%) identifying specific traits. While these characteristics varied, studies informed by similar theoretical underpinnings tended to focus on overlapping aspects. The interventions targeted either the intrinsic qualities of misinformation that increase its believability or the presentation tactics that enhance its spread, aiming to improve public literacy on these aspects. Approximately half of the interventions (14/30, 47%) did not report the specific characteristics of misinformation they targeted. Many of the studies (23/30, 77%) highlighted the use of theoretical underpinnings to develop their interventions, ranging from models focused on misinformation content to theories of learning and education. The principally used theoretical underpinnings were inoculation theory (building resistance to misinformation through methods such as promoting alternative explanations, fostering critical thinking, and enhancing digital literacy), motivational techniques, persuasive techniques, and frameworks addressing trust in medical information and professionals. Of the 30 studies, 6 (20%) did not report using theoretical underpinnings, raising questions about whether they were underreported or omitted entirely.

The publication time frames and intervention content frequently paralleled the trajectory of the COVID-19 pandemic, underscoring how health crises, despite their detrimental impacts, have accelerated the development and deployment of initiatives aimed at combating misinformation. However, 40% (12/30) of the papers targeted misinformation in broader health-related contexts, highlighting the persistent nature of misinformation and its dissemination both before and after the pandemic [61], driven by the ease of dissemination through online platforms and social media [62] as well as inherent human susceptibilities to misleading information [9]. The geographic representation among the studies overall was limited, which is significant because health misinformation varies across regions due to sociocultural influences that affect both the characteristics of misinformation and the ways populations engage with and disseminate information [63]. The WHO and the Center for Infectious Disease Research and Policy have postulated the critical role of cultural insights in developing effective public health strategies, highlighting the need for socioculturally appropriate strategies to influence health behaviors and perceptions [2,63]. As echoed in other systematic reviews, this could be a fruitful area of future research to better understand regional variations and tailor interventions accordingly [64].

The interventions used across the studies were diverse, including educational interventions, counter speech, inoculation games, and online distribution blockers. The educational interventions comprised courses, videos, text, or infographics. Although these were sometimes used independently, some of the studies (2/22, 9%) adopted a combined design that integrated both educational and counter-speech components, making it difficult to clearly distinguish the effects of each intervention type. Counter speech involves addressing misinformation by highlighting inaccuracies and providing correct information through techniques such as debunking, refuting, and correction frameworks [12]. Similarly, although inoculation games were sometimes used independently, they were often integrated into educational interventions and counter-speech strategies. This integration aligns with current literature, which often combines multiple intervention types to reinforce their impact and address the multifaceted nature of misinformation, providing benefits in timely correction of facts, while also enabling user literacy, education, and efficacy in recognizing misinformation in the long term.

Online intervention distribution blockers were defined as a distinct category during data extraction because of the notable rise in recent years in platform-led interventions and “technocognition” approaches such as algorithmic downranking, content moderation, advertisement blocking, and redirection. This review found that such strategies were not implemented in intervention designs. This gap is echoed in other systematic reviews that state the need for assessing the “effectiveness and safety of computer-driven corrective and interventional measures against health misinformation” [65], suggesting a fertile area for future research [66].

A gap highlighted in this review was the lack of interventions targeting specific populations, especially those considered more vulnerable and susceptible to misinformation, with most of the studies (22/30, 73%) defaulting to a “general population” target. A variety of human attributes were considered in the design and development of the interventions, categorized into three groups: (1) knowledge and information processing, (2) psychological and emotional influences, and (3) trust and social dynamics. These categories are neither exclusive nor entirely distinct due to the interconnected nature of human cognition, psychology, and behavior. Multiple studies (8/14, 57%) targeted several of these human attributes simultaneously. However, 16 (53%) of the 30 studies did not report or consider specific human attributes in the design of their interventions. Current literature [66] underscores the crucial role of these factors in combating misinformation, especially in public health crises, and studies reiterate the role of cognition, knowledge, biases, and emotional responses in the uptake and spread of misinformation; hence, understanding and addressing these human attributes is essential for designing effective interventions.

This generalization may be beneficial in some cases, considering the broad target populations of public health interventions. However, it is well noted in the literature that considering the interplay of human attributes that make populations susceptible to misinformation, such as emotional, educational, or social influence, is vital for intervention design. Understanding these factors, prioritizing interventions tailored to specific population groups, and comparing the impact of targeting different human attributes would provide insight into the most critical factors to address in intervention design, particularly for developing effective methodologies for populations experiencing more vulnerability.

The interventions broadly addressed either the intrinsic characteristics of misinformation that increase its believability or the presentation tactics that enhance its online dissemination, holistically aiming to improve audience digital literacy regarding the topic of misinformation. Commonly targeted elements included emotional language, medical conspiracies, reliance on fake experts, sensationalization, repetition, polarization, and discrediting credible sources. This aligns with the “characteristics of misinformation” framework proposed by Roozenbeek and van der Linden [60], although only 1 (3%) of the 30 included studies [48] implemented an intervention covering all these components. Interventionalists could benefit from a comprehensive approach that simultaneously addresses multiple characteristics of misinformation, given the current lack of studies exploring the interconnected nature of these factors.

Approximately half of the interventions (14/30, 47%) reviewed did not report the specific characteristics of misinformation they targeted. This raises questions about whether these characteristics were considered in the development of the interventions or whether the interventions were designed without such input. When characteristics are not explicitly addressed, interventions tend to focus on countering specific instances of misinformation rather than addressing the underlying characteristics that make misinformation believable. This approach may provide a short-term solution but is less effective in addressing the long-term problem. Current literature emphasizes the importance of fostering long-term strategies that foster critical thinking and resilience to misinformation through enhancing media literacy and cognitive processing skills in audiences [66]. By targeting intrinsic characteristics of misinformation and human attributes, interventions can be more sustainable and effective in the long term, which is a vital area with scope for further research, as supported by recent scoping reviews [64].

Definitions of misinformation, its intrinsic characteristics, and the population attributes that increase susceptibility and dissemination often lack clarity and uniformity across the reviewed studies. There is inconsistency in how misinformation is defined. This is reflected in how it is addressed through interventions and has been noted in other systematic reviews, which highlight that inconsistent definitions adversely affect the outcomes of interventional studies about health misinformation [64]. Many studies do not specify which characteristics of misinformation are the most problematic or which population attributes need to be addressed, limiting the specificity of intervention development. Although some of these factors are indirectly targeted through counter speech or educational courses, the lack of clear definitions reduces meaningful insight for interventionalists looking to develop more effective and specific interventions.

A significant proportion of the reviewed studies (23/30, 77%) highlighted the use of theoretical underpinnings to develop their interventions, ranging from models focused on misinformation content to learning and education theories. However, 6 (20%) of the 30 studies did not report any theoretical underpinnings. This omission raises concerns about whether they were excluded or not used at all, which is problematic, given the importance of such underpinnings in shaping effective interventions tailored to audience behavior and knowledge gaps, as noted in existing literature on digital interventions.

Inoculation theory was frequently used (6/30, 20%), involving preemptive exposure to weakened forms of misinformation to build resistance. This technique, known as “prebunking,” often included alerting participants to the fallacies underlying misinformation and drawing attention to its defining characteristics or digital patterns. Interventions incorporating debunking, correcting, and refutational strategies were also prominent (6/30, 20%). These methods were used either as didactic techniques, where misinformation was directly refuted, or in combination with inoculation strategies. The literature indicates significant variation in these approaches: traditional debunking involves underlining the bottom line or providing corrective explanations, while refutational debunking focuses on explaining why misinformation is incorrect, arguing that traditional debunking may not be as effective in addressing persistent misinformation. This theoretical debate is mirrored in noninterventional literature [66], which explores how psychological and cognitive mechanisms influence the correction of misinformation during infodemics, and continues to be an area for further research to determine which “debunking” technique is the most beneficial in the short and long terms.

Building future resistance to misinformation was a central theme in many of the included studies (15/30, 50%). Techniques such as promoting alternative explanations, fostering critical thinking, and enhancing digital literacy are supported by a broader body of research. Despite this, the interventions varied in their application, with some emphasizing psychological engagement and memory retention, while others focused on immediate behavioral changes. Many of the interventions (11/30, 37%) incorporated broader theoretical models, such as motivational techniques (eg, narratives and motivational interviewing to encourage behavior change), persuasive techniques (eg, nudge theory to promote mindful behavior regarding misinformation), and frameworks that addressed trust in medical information and professionals. Several of the studies lacked detailed explanations on how these theories were applied, implying a gap in the understanding of implementation science behind these frameworks.

In interventions that cited theoretical underpinnings for the learning and design components, the emphasis was on enhancing memory, cognitive processing, and engaging with digital content. While existing literature strongly supports the effectiveness of these strategies in educational contexts [67], their inconsistent reporting across the included studies highlights a broader issue: the need for systematic integration of theoretical underpinnings (including frameworks and models) to optimize misinformation interventions.

### Limitations

This scoping review has certain limitations. We only searched 5 databases, potentially missing relevant studies available elsewhere. Gray literature was excluded, potentially missing unpublished but implemented interventions. The review did not assess the outcomes or effectiveness of the interventions. Although our focus was on the design of the interventions, understanding their effectiveness could provide insights into successful strategies for combating misinformation in future research. Studies discussing online distribution blockers and relevant subcategories were excluded, as the review was limited to studies that specifically implemented these interventions. This may have resulted in missing information on the theoretical underpinnings and target characteristics or populations of these interventions. By limiting the review to online interventions, we may have overlooked effective offline strategies, especially important in regions with limited internet access. Finally, we included only English-language papers, unintentionally limiting the geographic scope and potentially introducing bias toward English-speaking contexts.

### Conclusions

This review offers valuable insights for health care professionals, policy makers, and public health organizations to design theory-informed interventions that counter health misinformation. The reviewed studies used various types of interventions, mainly educational courses (22/30, 73%), and they often combined counter speech and inoculation games, emphasizing the value of a multifaceted approach to combating health misinformation. Only a few studies (16/30, 53%) targeted specific misinformation traits, and gaps remain in identifying key contributors to the spread of misinformation in interventional studies, highlighting the need for future research to enhance intervention effectiveness. Many of the interventions (22/30, 73%) overlooked demographic targeting, limiting insight into susceptibility and missing opportunities to reach those who might benefit the most. Human attributes crucial in intervention designs were frequently underreported and more often reported in theoretical rather than practical contexts, indicating the need for collaboration between intervention developers, psychologists, and theorists. Theoretical underpinnings varied, with inconsistent reporting and interpretation, underscoring the need for consensus on definitions and the use of comprehensive frameworks. The review has highlighted inconsistencies in reporting the developmental principles and theoretical underpinnings of online interventions. More effective reporting is required, with explicit connections between the features of misinformation and outcomes in terms of intervention development and design. At present, the reporting gaps limit the ability to holistically analyze how development approaches affect the performance and efficacy of such interventions.

## Appendix

Multimedia Appendix 1 Sample electronic search strategies.

Multimedia Appendix 2 Data extraction form.

Multimedia Appendix 3 Summary of study features, intervention types, and targeted misinformation characteristics.

Multimedia Appendix 4 Health misinformation ScR dataset.

Multimedia Appendix 5 Preferred Reporting Items for Systematic reviews and Meta-Analyses extension for Scoping Reviews (PRISMA-ScR) Checklist.

## Abbreviations

MeSH: Medical Subject Headings
PRISMA-ScR: Preferred Reporting Items for Systematic Reviews and Meta-Analyses extension for Scoping Reviews
WHO: World Health Organization

## Data Availability

The datasets extracted and analyzed in this review are available in Multimedia Appendix 4.
