# Supplementary material for: Online Interventions Addressing Health Misinformation: Scoping Review
Source: J Med Internet Res. 2025 Sep 4;27:e69618. doi: 10.2196/69618 (PMC12447009; doi:10.2196/69618)
Supplement: Multimedia Appendix 1 [file jmir_v27i1e69618_app1.docx]

**Multimedia appendix 1 – Sample electronic search strategies**

**Database: PubMed**

(("misinformation*"[Title/Abstract] OR "disinformation"[MeSH Terms] OR ("disinformation"[MeSH Terms] OR "disinformation"[All Fields] OR ("fake"[All Fields] AND "news"[All Fields]) OR "fake news"[All Fields]) OR (("false"[All Fields] OR "falsely"[All Fields]) AND ("news"[Publication Type] OR "news"[All Fields]))) AND ("delivery of health care"[MeSH Terms] OR ("delivery"[All Fields] AND "health"[All Fields] AND "care"[All Fields]) OR "delivery of health care"[All Fields] OR "healthcare"[All Fields] OR "healthcare s"[All Fields] OR "healthcares"[All Fields] OR ("health"[MeSH Terms] OR "health"[All Fields] OR "health s"[All Fields] OR "healthful"[All Fields] OR "healthfulness"[All Fields] OR "healths"[All Fields])) AND ("internet based intervention"[MeSH Terms] OR ("internet based"[All Fields] AND "intervention"[All Fields]) OR "internet based intervention"[All Fields] OR ("internet"[All Fields] AND "based"[All Fields] AND "intervention"[All Fields]) OR "internet based intervention"[All Fields] OR ("internet based intervention"[MeSH Terms] OR ("internet based"[All Fields] AND "intervention"[All Fields]) OR "internet based intervention"[All Fields] OR ("online"[All Fields] AND "intervention"[All Fields]) OR "online intervention"[All Fields]) OR ("internet based intervention"[MeSH Terms] OR ("internet based"[All Fields] AND "intervention"[All Fields]) OR "internet based intervention"[All Fields] OR ("web"[All Fields] AND "based"[All Fields] AND "intervention"[All Fields]) OR "web based intervention"[All Fields]) OR ("internet based intervention"[MeSH Terms] OR ("internet based"[All Fields] AND "intervention"[All Fields]) OR "internet based intervention"[All Fields] OR ("internet"[All Fields] AND "intervention"[All Fields]) OR "internet intervention"[All Fields]) OR ("early intervention, educational"[MeSH Terms] OR ("early"[All Fields] AND "intervention"[All Fields] AND "educational"[All Fields]) OR "educational early intervention"[All Fields] OR ("early"[All Fields] AND "intervention"[All Fields]) OR "early intervention"[All Fields]))) AND ((y_5[Filter]) AND (english[Filter]))
