# Supplementary material for: Online Interventions Addressing Health Misinformation: Scoping Review
Source: J Med Internet Res. 2025 Sep 4;27:e69618. doi: 10.2196/69618 (PMC12447009; doi:10.2196/69618)
Supplement: Multimedia Appendix 2 [file jmir_v27i1e69618_app2.docx]

**Multimedia Appendix 2 – Data Extraction Tool**

Misinformation Extraction Tool 0.5

* Required

1. Paper Title *
2. Author Name *
3. Paper DOI *
4. Year of Publication *
5. Reviewer Name *

Reviewer 1

Reviewer 2

1. Include or Exclude *

Include

Exclude

1. State the reason for exclusion

Reasons could include: offline, not interventional design, not addressing health misinformation

1. Type of intervention * Educational courses

Counterspeech

Inoculation games

Online intervention distribution blockers

Other

1. If **"educational courses"** selected - describe

For example - Written learning modules/ courses, video learning modules, online peer learning, discussion platforms, fact-checking training

1. If **"counterspeech"** selected - please specify

For example - myth debunking, fact checking, expert testimonials, personal stories, humour, satire, call to action, questioning source credibility, encouraging critical thinking, highlighting consequences, etc...

1. If **"inoculation games"** selected - specify

For example - debate games, simulation games, identification games, interactive narrative games, persuasion detection games, cognitive bias training games, etc...

1. If **"online intervention distribution blockers"** selected - specify

For example - Ad blockers, content filters, social medial blockers, email spam blockers, content blocking DNS (domain name system) serves, comment moderation tools, etc...

1. If **"other**" selected - specify
2. Describe the intervention involved *
3. What was the nature of the intervention's content *

Text

Multimedia (eg. videos, audios, images)

Not mentioned/ not applicable

Other

1. What was the mode of delivery of the intervention *

SMS

E-mail

Websites

Social media

Other

1. If social media was used for delivery, what was the principal platform?

Facebook

Instagram

Whatsapp

Snapchat

LinkedIn

Twitter/X

Tiktok

Reddit

Discord

None Mentioned/ Not Applicable

Other

1. Demographic age of the intervention's target population

Children <18

Young adults 18-24

Adults 25-64

Senior citizens ≥65

Not mentioned/ Not applicable

1. Demographic type of the intervention's target population

Healthcare workers

Patients of specific disease

Educators

Institutional leadership

Carers

General public

Students

Not mentioned/ not applicable

Other

1. Describe the type of demographic targeted by the intervention - if included

For example - vulnerable populations, underprivileged individuals, literacy levels, educators, healthcare professionals etc...

1. Describe the socio-economic status of the demographic - if included
2. Describe the nationality of the target demographic - if included
3. Describe the setting or target country/region of the study - if included
4. What type of susceptibility characteristic of the target population is being targeted by the intervention, if mentioned

Examples: targeting illusory truth, cognitive biases, pre-risk exposure, education levels

1. Was a framework or method mentioned as the basis of the intervention? *

Yes

No

1. Describe the type of framework or method used to guide the intervention - if mentioned
2. What characteristic of misinformation is being targeted by the intervention? *

Emotional language

Medical conspiracies

Medical Jargon

Sensationalization

Fake experts

Vagueness

Polarisation

None mentioned

Other

1. Describe the characteristics of misinformation being targeted by the intervention
