# Supplementary material for: Online Interventions Addressing Health Misinformation: Scoping Review
Source: J Med Internet Res. 2025 Sep 4;27:e69618. doi: 10.2196/69618 (PMC12447009; doi:10.2196/69618)
Supplement: Multimedia Appendix 3 [file jmir_v27i1e69618_app3.docx]

**Multimedia Appendix 3 – Metatable of Results**

| **Study Name** | **Author Name** | **Year** | **Location** | **Study Design** | **Type of Intervention** | **Presence of Frameworks** | **Content framework** | **Design framework used** | **Content of misinformation** | **Design content of intervention** | **Mode of Delivery** | **Characteristics of misinformation** | **Demographic Groups** | **Human attributes** |
| --- | --- | --- | --- | --- | --- | --- | --- | --- | --- | --- | --- | --- | --- | --- |
| **“A Friendly Conversation.” Developing an eHealth Intervention to Increase COVID-19 Testing and Vaccination Literacy Among Women with Criminal and Legal System Involvement**[1] | Geana et Al | 2023 | United States of America | Single Arm Pre-text/Post-test | Educational Courses | Present |  | Reflective model of aesthetic experience; Cognitive theory of multi-media learning; Social identity theory | COVID-19 | Multi-media | Websites; Web based eHealth application |  | Women with criminal and legal system involvement | Health literacy levels; resistance to authoritative health messages/ sources; Social determinants of Health |
| **A Web-Based Public Health Intervention for Addressing Vaccine Misinformation: Analysis of Learner Engagement and Shift in Hesitancy to Vaccinate**[2] | Powell et Al | 2023 | N/A | Single Arm Pre-text/Post-test "quasi study" | Educational Courses | Present |  | Gamification theory | COVID-19 | Multi-media; Text | Websites; Online course development platform |  | General public/ not specified |  |
| **Addressing Antivaccine Sentiment on Public Social Media Forums Through Web-Based Conversations Based on Motivational Interviewing Techniques: Observational Study**[3] | Scales et Al | 2023 | United States of America | Single arm post-test study | Counterspeech | Present | Inoculation theory; Motivational Interviweing; Narratives; Promoting critical thinking |  | COVID-19 | Text | Social Media; Facebook |  | General public/ not specified | Illusory truth |
| **Addressing myths and vaccine hesitancy: A randomised trial**[4] | Steffens et Al[4] | 2021 | Australia | Randomised control tiral | Counterspeech | Present | Deubking/refutation/correction |  | Other health misinformation | Text | Websites; Web-based/online questionnaire/survey | Medical conspiracies; Repetition | Parents | Familiarity bias |
| **Can touch this: training to correct police officer beliefs about overdose from incidental contact with fentanyl**[5] | Del Pozo et Al | 2021 | United States of America | Single Arm Pre-text/Post-test | Counterspeech | Absent |  |  | Other health misinformation | Multi-media | Zoom Video Communications Inc | Medical conspiracies | Police officers |  |
| **Combating misinformation: The effects of infographics in verifying false vaccine news** [6] | Dommgaard et Al | 2021 | United States of America | Randomised control tiral | Educational Courses | Present |  | Processing fluencey theory | Other health misinformation | Multi-media | Web-based/online questionnaire/ survey | Emotional language; sensationalisation; fake experts; Outdated information; grammatical and spelling errors; vagueness | General public/ not specified |  |
| **Correcting vaccine misinformation: A failure to replicate familiarity or fear-driven backfire effects**[7] | Ecker et Al | 2023 | United Kingdom | Randomised control tiral | Educational Courses | Present | Familiarity boost theory; Appealing to fear and emotions |  | Other health misinformation | Multi-media; Text | Web-based/online questionnaire/ survey |  | General public/ not specified | Continued influence effect; Familiarity bias; Illusory truth |
| **Debunking highly prevalent health misinformation using audio dramas delivered by WhatsApp: evidence from a randomised controlled trial in Sierra Leone**[8] | Winters et Al | 2021 | Sierra Leone | Randomised control tiral | Educational Courses | Present | Deubking/refutation/correction; Plausible alternative framework; Avoiding misinformation framework |  | Other health misinformation | Multi-media | Social Media; Whatsapp |  | General public/ not specified | Continued influence effect; Repeated exposure; Cognitive biases |
| **Does public fear that bats spread COVID-19 jeopardize bat conservation?**[9] | Lu et Al | 2021 | China | Randomised control tiral | Educational Courses | Absent |  |  | COVID-19 | Multi-media; Text | Social Media; WeChat | Emotional language; sensationalisation; medical conspiracies; grammartical and syntax changes | General public/ not specified | Pre-intervention knowledge levels |
| **Educational Video Intervention to Improve Health Misinformation Identification on WhatsApp Among Saudi Arabian Population: Pre-Post Intervention Study**[10] | Alsaad et Al | 2024 | Saudi Arabia | Single Arm Pre-text/Post-test | Educational Courses | Present | Inoculation theory | Cognitive theory of multi-media learning; Message interpretation process theory | General Misinformation | Multi-media | Web-based/online questionnaire/ survey | Presentation of misinformation | General public/ not specified | Health literacy levels; Emotive manipulation; Rumour spreading behaviour |
| **Effects of an Online Community Peer-support Intervention on COVID-19 Vaccine Misinformation Among Essential Workers: Mixed-methods Analysis**[11] | Ugarte et Al | 2023 | United States of America | Randomised control tiral | Educational Courses; Counterspeech | Absent |  |  | COVID-19 | Multi-media; Text | Social Media; Facebook | Stigma; Politicisation | Educators; Healthcare workers; |  |
| **Effects of Narrative Messages on Key COVID-19 Protective Responses: Findings From a Randomized Online Experiment**[12] | Iles et Al | 2022 | United States of America | Randomised control tiral | Educational Courses | Present | Narratives |  | COVID-19 | Text | Web-based/online questionnaire/ survey | Medical conspiracies | General public/ not specified | Emotive manipulation; Familiarity bias; resistance to authoritative health messages/source; Erroding trust in social institutions and experts; Illusory truth |
| **Empathic Engagement With the COVID-19 Vaccine Hesitant in Private Facebook Groups: A Randomized Trial**[13] | Abroms et Al | 2023 | United States of America | Single arm post-test study | Counterspeech | Present | Deubking/refutation/correction; Fuzzy trace theory; Empathetic engagement |  | COVID-19 | Multi-media; Text | Social Media; Facebook |  | Non Vaccinated Individuals |  |
| **Empathic Engagement with the Vaccine Hesitant in Online Spaces**[14] | Koban et Al | 2023 | United States of America | Randomised control tiral | Counterspeech | Present | Fuzzy trace theory |  | COVID-19 | Text | Social Media; Facebook | De-contextualised verbatim information (facts, statistics) | General public/ not specified |  |
| **Evaluating the impact of short animated videos on COVID-19 vaccine hesitancy: An online randomized controlled trial**[15] | Beleites et Al | 2024 | United States of America | Randomised control tiral | Educational Courses | Absent |  |  | COVID-19 | Multi-media; Text | Websites |  | General public/ not specified |  |
| **Evaluation of a template for countering misinformation—Real-world Autism treatment myth debunking[16]** | Paynter et Al | 2019 | Australia | Randomised control tiral | Educational Courses | Present | Deubking/refutation/correction [against debunking] |  | Other health misinformation | Multi-media; Text | Websites | Emotional language; Fake experts; Medical conspiracies; Celebrity endorsement; Repetition | Carers; Educators; Healthcare  workers | Continued influence effect; Emotive manipulation; Repeated exposure |
| **Fighting COVID-19 Misinformation on Social Media: Experimental Evidence for a Scalable Accuracy-Nudge Intervention[17]** | Pennycook et Al | 2020 | United States of America | Randomised control tiral | Educational Courses | Present | Inattention-based account of misinformation sharing; Nudge theory |  | COVID-19 | Multi-media; Text | Web-based/online questionnaire/ survey | Partisan alignment/distraction | General public/ not specified |  |
| **Fighting COVID-19 Misinformation through an Online Game Based on the Inoculation Theory: Analyzing the Mediating Effects of Perceived Threat and Persuasion Knowledge[18]** | Ma et Al | 2023 | United States of America | Randomised control tiral | Inoculation Game | Present | Inoculation theory; Persuasion knowledge |  | COVID-19 | Multi-media; Text | Web-based/online questionnaire/ survey | Emotional language; Fake experts; Medical conspiracies; Polarisation; Discredit; Rolling; Spreading of false knowledge | General public/ not specified |  |
| **Fighting the Spread of COVID-19 Misinformation in Kyrgyzstan, India, and the United States: How Replicable Are Accuracy Nudge Interventions?[19]** | Gavin et Al | 2022 | United states of America; India; Kyrgyzstan | Randomised control tiral | Educational Courses | Present | Promoting critical thinking; Nudge theory; Cognitive function; Dual processing theory |  | COVID-19 | Multi-media; Text | Web-based/online questionnaire/ survey | Ease of dissemination | General public/ not specified | Cognitive biases; System 1 thinking |
| **HPV Vaccination Champions: Evaluating a Technology-Mediated Intervention for Parents[20]** | Sundstorm et Al | 2021 | United States of America | Single Arm Pre-text/Post-test | Educational Courses; Counterspeech | Absent |  |  | Other health misinformation | Multi-media; Text; Focus groups; Webinars | Social Media; Facebook; E-mail |  | Parents |  |
| **Inoculation works and health advocacy backfires: Building resistance to COVID-19 vaccine misinformation in a low political trust context[21]** | Jiang et Al | 2022 | Hong Kong | Randomised control tiral | Educational Courses | Present | Inoculation theory |  | COVID-19 | Text | Web-based/online questionnaire/ survey |  | General public; Students |  |
| **Intervening on Trust in Science to Reduce Belief in COVID-19 Misinformation and Increase COVID-19 Preventive Behavioral Intentions: Randomized Controlled Trial[22]** | Agley et Al | 2021 | United States of America | Randomised control tiral | Educational Courses | Present | Trust in science and scientists |  | COVID-19 | Multi-media | Web-based/online questionnaire/ survey | Medical conspiracies | General public/ not specified | Trust in science |
| **Investigating and Improving the Accuracy of US Citizens' Beliefs About the COVID-19 Pandemic: Longitudinal Survey Study[23]** | Stekelenburg et Al | 2021 | United States of America | Randomised control tiral | Educational Courses | Present | Promoting critical thinking; Gateway belief model; Boosting approach |  | COVID-19 | Multi-media; Infographics | Web-based/online questionnaire/ survey |  | General public/ not specified | Health literacy levels |
| **Misinformation in nutrition through the case of coconut oil: An online before-and-after study Author links open overlay panel[24]** | Durate et Al | 2022 | Brazil | Single Arm Pre-text/Post-test | Counterspeech | Absent |  |  | Other health misinformation | Text | Web-based/online questionnaire/ survey |  | General public; Students |  |
| **Refuting Spurious COVID-19 Treatment Claims Reduces Demand and Misinformation Sharing [25]** | MacFarlane et Al | 2021 | United States of America | Randomised control tiral | Counterspeech | Present | Deubking/refutation/correction; Highlighting discrepancies between false and factual information |  | Other health misinformation | Text | Web-based/online questionnaire/ survey | Fake experts; medical conspiracies; Polarisation; discredit; Illusion of causality; Appeal to morality; Overlooking risks; Appeal to nature | General public/ not specified | Cognitive biases; pre-existing beliefs |
| **Seeking Formula for Misinformation Treatment in Public Health Crises: The Effects of Corrective Information Type and Source[26]** | Van der Meer et Al | 2019 | United States of America | Randomised control tiral | Educational Courses | Present | Deubking/refutation/correction |  | Other health misinformation | Text | Websites |  | General public/ not specified |  |
| **Testing the Efficacy of Attitudinal Inoculation Videos to Enhance COVID-19 Vaccine Acceptance: Quasi-Experimental Intervention Trial [27]** | Piltch-Loeb et Al | 2022 | United States of America | Randomised control tiral "Quasi" | Educational Courses | Present | Inoculation theory |  | COVID-19 | Multi-media | Web-based/online questionnaire/ survey | Emotional language; Medical conspiracies | General public/ not specified |  |
| **The Effect of a Wordless, Animated, Social Media Video Intervention on COVID-19 Prevention: Online Randomized Controlled Trial[28]** | Vandormael et Al | 2021 | United States of America, Mexico, UK, Germany, Spain | Randomised control tiral | Educational Courses | Absent |  |  | COVID-19 | Multi-media | Websites |  | General public/ not specified |  |
| **The effects of a news literacy video and real-time corrections to video misinformation related to sunscreen and skin cancer.[29]** | Vraga et Al | 2021 | United States of America | Randomised control tiral | Educational Courses | Present | Inoculation theory, Debunking/refutation/correction, News Literacy |  | Other health misinformation | Multi-media; Text | Websites |  | General public/ not specified |  |
| **The usefulness of a checklist approach-based confirmation scheme in identifying unreliable COVID-19-related health information: a case study in Japan[30]** | Tanemura et Al | 2022 | Japan | Single Arm Pre-text/Post-test | Educational Courses | Present | Mindsponge mechanism theory; Confirmation scheme |  | COVID-19 | Text | Websites; Web-based/online questionnaire/survey | Five step framework - confirmation theory | General public/ not specified | Health literacy levels |

**References:**

[1] M. v. Geana, P. Liu, J. Pei, S. Anderson, and M. Ramaswamy, ‘“A Friendly Conversation.” Developing an ehealth intervention to increase COVID-19 testing and vaccination literacy among women with criminal and legal system involvement.’, *J. Health Commun.*, vol. 29, no. 2, pp. 131–142, 2024, doi: 10.1080/10810730.2023.2293094.

[2] L. Powell, R. Nour, Y. Zidoun, S. Kaladhara, H. Al Suwaidi, and N. Zary, ‘A Web-Based Public Health Intervention for Addressing Vaccine Misinformation: Protocol for Analyzing Learner Engagement and Impacts on the Hesitancy to Vaccinate’, *JMIR Res. Protoc.*, vol. 11, no. 5, p. e38034, May 2022, doi: 10.2196/38034.

[3] D. Scales *et al.*, ‘Addressing Antivaccine Sentiment on Public Social Media Forums Through Web-Based Conversations Based on Motivational Interviewing Techniques: Observational Study’, *JMIR Infodemiology*, vol. 3, p. e50138, Nov. 2023, doi: 10.2196/50138.

[4] M. Steffens, A. Dunn, M. Marques, M. Danchin, H. Witteman, and J. Leask, ‘Addressing Myths and Vaccine Hesitancy: a Randomized Trial’, *Pediatrics*, vol. 148, no. 5, 2021, doi: 10.1542/peds.2020-049304.

[5] B. Del Pozo, E. Sightes, S. Kang, J. Goulka, B. Ray, and L. A. Beletsky, ‘Can touch this: training to correct police officer beliefs about overdose from incidental contact with fentanyl’, *Health Justice*, vol. 9, no. 1, p. 34, Nov. 2021, doi: 10.1186/s40352-021-00163-5.

[6] S. Domgaard and M. Park, ‘Combating misinformation: the effects of infographics in verifying false vaccine news’, *Health Educ. J.*, vol. 80, no. 8, p. 974‐986, 2021, doi: 10.1177/00178969211038750.

[7] U. K. H. Ecker, C. X. M. Sharkey, and B. Swire-Thompson, ‘Correcting vaccine misinformation: A failure to replicate familiarity or fear-driven backfire effects.’, *PLoS ONE*, vol. 18, no. 4, 2023, doi: 10.1371/journal.pone.0281140.

[8] M. Winters *et al.*, ‘Debunking highly prevalent health misinformation using audio dramas delivered by WhatsApp: evidence from a randomised controlled trial in Sierra Leone’, *BMJ Glob. Health*, vol. 6, no. 11, p. e006954, Nov. 2021, doi: 10.1136/bmjgh-2021-006954.

[9] M. Lu *et al.*, ‘Does public fear that bats spread COVID-19 jeopardize bat conservation?’, *Biol. Conserv.*, vol. 254, p. 108952, Feb. 2021, doi: 10.1016/j.biocon.2021.108952.

[10] E. Alsaad and S. AlDossary, ‘Educational Video Intervention to Improve Health Misinformation Identification on WhatsApp Among Saudi Arabian Population: Pre-Post Intervention Study’, *JMIR Form. Res.*, vol. 8, p. e50211, Jan. 2024, doi: 10.2196/50211.

[11] D. Ugarte and S. Young, ‘Effects of an Online Community Peer-support Intervention on COVID-19 Vaccine Misinformation Among Essential Workers: mixed-methods Analysis’, *West. J. Emerg. Med.*, vol. 24, no. 2, p. 264‐268, 2023, doi: 10.5811/westjem.2023.1.57253.

[12] I. Iles, A. Gaysynsky, and W. Sylvia Chou, ‘Effects of Narrative Messages on Key COVID-19 Protective Responses: findings From a Randomized Online Experiment’, *Am. J. Health Promot.*, vol. 36, no. 6, p. 934‐947, 2022, doi: 10.1177/08901171221075612.

[13] L. C. Abroms *et al.*, ‘Empathic Engagement With the COVID-19 Vaccine Hesitant in Private Facebook Groups: A Randomized Trial’, *Health Educ. Behav. Off. Publ. Soc. Public Health Educ.*, p. 10901981231188313, Jul. 2023, doi: 10.1177/10901981231188313.

[14] D. D. Koban, ‘Empathic engagement with the vaccine hesitant in online spaces.’, vol. 84, no. 9-A, p. No Pagination Specified-No Pagination Specified, 2023.

[15] F. Beleites *et al.*, ‘Evaluating the impact of short animated videos on COVID-19 vaccine hesitancy: An online randomized controlled trial’, *Internet Interv.*, vol. 35, p. 100694, Mar. 2024, doi: 10.1016/j.invent.2023.100694.

[16] J. Paynter *et al.*, ‘Evaluation of a template for countering misinformationâ€”Real-world Autism treatment myth debunking’, *PloS One*, vol. 14, no. 1, 2019, doi: 10.1371/journal.pone.0210746.

[17] G. Pennycook, J. McPhetres, Y. Zhang, J. G. Lu, and D. G. Rand, ‘Fighting COVID-19 misinformation on social media: Experimental evidence for a scalable accuracy-nudge intervention.’, *Psychol. Sci.*, vol. 31, no. 7, pp. 770–780, 2020, doi: 10.1177/0956797620939054.

[18] J. Ma, Y. Chen, H. Zhu, and Y. Gan, ‘Fighting COVID-19 Misinformation through an Online Game Based on the Inoculation Theory: analyzing the Mediating Effects of Perceived Threat and Persuasion Knowledge’, *Int. J. Environ. Res. Public. Health*, vol. 20, no. 2, 2023, doi: 10.3390/ijerph20020980.

[19] L. Gavin, J. McChesney, A. Tong, J. Sherlock, L. Foster, and S. Tomsa, ‘Fighting the spread of COVID-19 misinformation in Kyrgyzstan, India, and the United States: How replicable are accuracy nudge interventions?’, *Technol. Mind Behav.*, vol. 3, no. 3, p. No Pagination Specified-No Pagination Specified, 2022, doi: 10.1037/tmb0000086.

[20] B. Sundstrom *et al.*, ‘HPV Vaccination Champions: Evaluating a Technology-Mediated Intervention for Parents’, *Front. Digit. Health*, vol. 3, p. 636161, 2021, doi: 10.3389/fdgth.2021.636161.

[21] L. C. Jiang, M. Sun, T. H. Chu, and S. C. Chia, ‘Inoculation works and health advocacy backfires: Building resistance to COVID-19 vaccine misinformation in a low political trust context.’, *Front. Psychol.*, vol. 13, 2022, doi: 10.3389/fpsyg.2022.976091.

[22] J. Agley, Y. Xiao, E. E. Thompson, X. Chen, and L. Golzarri-Arroyo, ‘Intervening on Trust in Science to Reduce Belief in COVID-19 Misinformation and Increase COVID-19 Preventive Behavioral Intentions: Randomized Controlled Trial’, *J. Med. Internet Res.*, vol. 23, no. 10, p. e32425, Oct. 2021, doi: 10.2196/32425.

[23] A. van Stekelenburg, G. Schaap, H. Veling, and M. Buijzen, ‘Investigating and Improving the Accuracy of US Citizens’ Beliefs About the COVID-19 Pandemic: Longitudinal Survey Study’, *J. Med. Internet Res.*, vol. 23, no. 1, p. e24069, Jan. 2021, doi: 10.2196/24069.

[24] A. C. Duarte *et al.*, ‘Misinformation in nutrition through the case of coconut oil: An online before-and-after study’, *Nutr. Metab. Cardiovasc. Dis. NMCD*, vol. 32, no. 6, pp. 1375–1384, Jun. 2022, doi: 10.1016/j.numecd.2022.02.002.

[25] D. MacFarlane, L. Q. Tay, M. J. Hurlstone, and U. K. H. Ecker, ‘Refuting spurious COVID-19 treatment claims reduces demand and misinformation sharing.’, *J. Appl. Res. Mem. Cogn.*, vol. 10, no. 2, pp. 248–258, 2021, doi: 10.1037/h0101793.

[26] T. G. L. A. van der Meer and Y. Jin, ‘Seeking formula for misinformation treatment in public health crises: The effects of corrective information type and source.’, *Health Commun.*, vol. 35, no. 5, pp. 560–575, 2020, doi: 10.1080/10410236.2019.1573295.

[27] R. Piltch-Loeb *et al.*, ‘Testing the Efficacy of Attitudinal Inoculation Videos to Enhance COVID-19 Vaccine Acceptance: Quasi-Experimental Intervention Trial’, *JMIR Public Health Surveill.*, vol. 8, no. 6, p. e34615, Jun. 2022, doi: 10.2196/34615.

[28] V *et al.*, ‘The {Effect} of a {Wordless}, {Animated}, {Social} {Media} {Video} {Intervention} on {COVID}-19 {Prevention}: online {Randomized} {Controlled} {Trial}’, *JMIR Public Health Surveill.*, vol. 7, no. 7, p. e29060, 2021, doi: 10.2196/29060.

[29] E. K. Vraga, L. Bode, and M. Tully, ‘The effects of a news literacy video and real-time corrections to video misinformation related to sunscreen and skin cancer.’, *Health Commun.*, vol. 37, no. 13, pp. 1622–1630, 2022, doi: 10.1080/10410236.2021.1910165.

[30] N. Tanemura and T. Chiba, ‘The usefulness of a checklist approach-based confirmation scheme in identifying unreliable COVID-19-related health information: a case study in Japan’, *Humanit. Soc. Sci. Commun.*, vol. 9, no. 1, p. 270, 2022, doi: 10.1057/s41599-022-01293-3.
